# BiVO_4_ Photoanode with Exposed (040) Facets for Enhanced Photoelectrochemical Performance

**DOI:** 10.1007/s40820-017-0163-3

**Published:** 2017-10-31

**Authors:** Ligang Xia, Jinhua Li, Jing Bai, Linsen Li, Shuai Chen, Baoxue Zhou

**Affiliations:** 1 School of Environmental Science and Engineering, Shanghai Jiao Tong University, No. 800 Dongchuan Rd, Shanghai, 200240 People’s Republic of China; 2 Key Laboratory of Thin Film and Microfabrication Technology, Ministry of Education, Shanghai, 200240 People’s Republic of China

**Keywords:** BiVO_4_ photoanode, Photoelectrochemical, Water splitting, Organic pollutant degradation

## Abstract

A BiVO_4_ photoanode with exposed (040) facets was prepared to enhance its photoelectrochemical performance. The exposure of the (040) crystal planes of the BiVO_4_ film was induced by adding NaCl to the precursor solution. The as-prepared BiVO_4_ photoanode exhibits higher solar-light absorption and charge-separation efficiency compared to those of an anode prepared without adding NaCl. To our knowledge, the photocurrent density (1.26 mA cm^−2^ at 1.23 V vs. RHE) of as-prepared BiVO_4_ photoanode is the highest according to the reports for bare BiVO_4_ films under simulated AM1.5G solar light, and the incident photon-to-current conversion efficiency is above 35% at 400 nm. The photoelectrochemical (PEC) water-splitting performance was also dramatically improved with a hydrogen evolution rate of 9.11 μmol cm^−2^ h^−1^, which is five times compared with the BiVO_4_ photoanode prepared without NaCl (1.82 μmol cm^−2^ h^−1^). Intensity-modulated photocurrent spectroscopy and transient photocurrent measurements show a higher charge-carrier-transfer rate for this photoanode. These results demonstrate a promising approach for the development of high-performance BiVO_4_ photoanodes which can be used for efficient PEC water splitting and degradation of organic pollutants.

**Electronic supplementary material:**

The online version of this article (doi:10.1007/s40820-017-0163-3) contains supplementary material, which is available to authorized users.

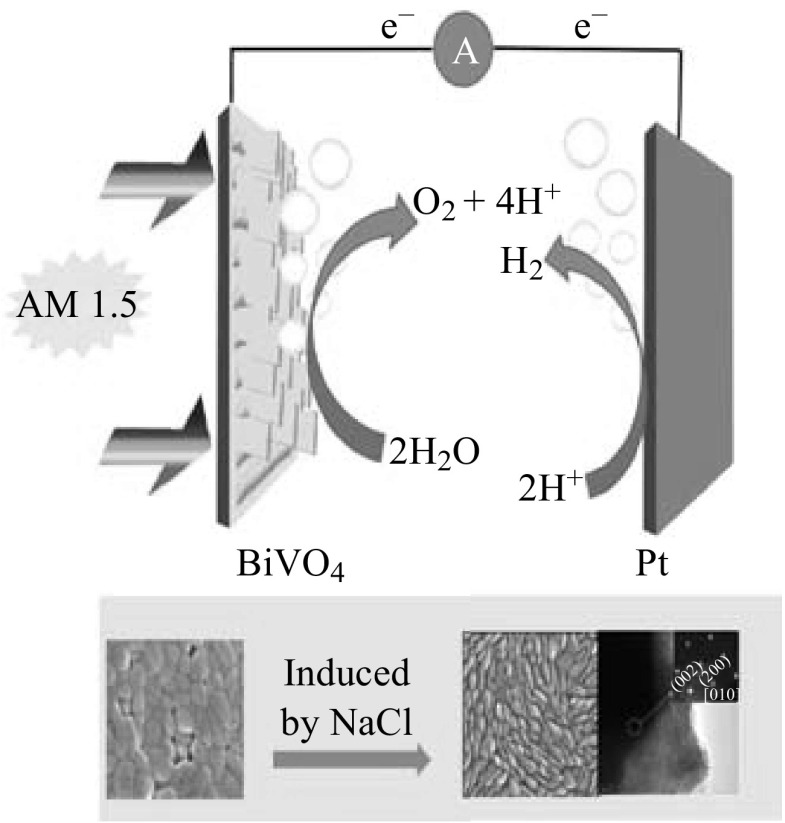

## Highlights

- A BiVO_4_ photoanode with exposed (040) facets was prepared by an improved chemical bath deposition method, where NaCl was used to induce the exposure of (040) facets
- The photoelectrochemical performance of as-synthesized BiVO_4_ photoanode with exposed (040) facets was strongly enhanced compared to that photoanode without exposure of (040) facets

## Introduction

Overuse of fossil fuels has resulted in serious environmental problems, and global energy shortages have become an increasingly urgent issue. Consequently, clean and renewable energy sources such as solar energy have become an attractive way to address this energy crisis. Photoelectrochemical (PEC) water splitting and organic pollutant degradation driven by visible light have attracted worldwide attention as promising applications of solar energy [1–4]. However, a key technical problem faced in PEC application is the development of suitable photoelectrodes, which play an important role in the PEC conversion of solar energy to chemical energy. Among the various photoelectrodes studied for PEC applications, monoclinic bismuth vanadate (BiVO_4_) is considered to be a promising material owing to its moderate band gap (~2.4 eV) and appropriate band-edge positions, which allow it to absorb as much as 11% of the solar spectrum [5–7]. However, the photocatalytic activity of bare BiVO_4_ is still not ideal for practical applications because of its excessive charge recombination, poor charge transport, and slow oxidation kinetics. To address these issues, several strategies have been developed to improve the photoelectrochemical activity of BiVO_4_, such as doping with foreign elements (e.g., Mo and W) [8–10], reduction to create oxygen vacancies [11–13], and coupling with co-catalysts (e.g., FeOOH and Co–Pi) [14–16] or other semiconductors to form stable heterojunction photoanodes [17–22].

Recently, it has been reported that the crystal plane structure of a semiconductor material can influence its photocatalytic and PEC activities. For example, powdered BiVO_4_ with exposed (040) facets shows enhanced photocatalytic activity [23, 24], and TiO_2_ photoanodes with highly energetic exposed (001) facets [25] and WO_3_ photoanodes with highly reactive exposed (002) facets [26] exhibit enhanced PEC activity in water splitting and organic pollutant degradation. Thus, it is easy to conclude that BiVO_4_ photoanodes with exposed active facets will show enhanced PEC properties. However, there are few reports on BiVO_4_ photoanodes with exposed (040) facets, and the preparation of such BiVO_4_ photoanodes remains a challenge. Generally, (040) facets are located on the lateral side and are difficult to expose because the adjacent facets grow together easily to form a compact morphology.

NaCl, one of the most common salts, has been used to regulate the growth of TiO_2_, SnO_2_, and CH_3_NH_3_PbI_3_ crystals [27–29]. The presence of chlorine ions in a solution can change its ionic strength and the coordinating ability of species therein, and chlorine ions can be easily adsorbed onto specific crystal facets, regulating the crystal growth. Therefore, we supposed that chlorine ions would adsorb on the (040) plane of BiVO_4_ and form a diffuse barrier to crystal growth, thereby leading to the exposure of more (040) facets. Furthermore, the chlorine ions may reduce the formation of BiVO_4_ by changing the composition or coordinating structure of the growing unit; consequently, the crystallization process becomes slower and easier to control.

The hydrothermal method is generally applied to prepare BiVO_4_ films with exposed (040) facets [30]. However, this method requires an autoclave to generate the super-critically high pressures required and is not suitable for large-scale production. Alternatively, the chemical bath deposition (CBD) method is cheap and easy to scale-up for industrial production. Furthermore, the CBD method has been used to prepare microcrystalline BiVO_4_ films on fluorine-doped tin oxide (FTO) substrates by Luo [31].

In the present study, a BiVO_4_ photoanode with exposed (040) facets is prepared, using NaCl to induce the exposure of the (040) facets. The results show that the BiVO_4_ photoanode prepared by the improved CBD method has one of the highest current densities reported for unmodified BiVO_4_ films.

## Experimental

### Preparation of BiVO_4_ Photoanode

The synthetic procedure for the BiVO_4_ photoanode was modified from that reported by Luo et al. [31]. For deposition of the BiVO_4_ seed layer on the FTO substrate, 5 mmol Bi(NO_3_)_3_·5H_2_O and 5 mmol NH_4_VO_3_ were dissolved in 15 mL of 23.3 wt% HNO_3_ aqueous solution, followed by the addition of 7.5 mL (5 g/100 mL) polyvinyl alcohol and 5 mmol citric acid. The solution was dropped and spin-coated onto a clean FTO substrate followed by heat treatment at 450 °C for 4 h. As a typical procedure to prepare BiVO_4_, 5.025 g ethylenediaminetetraacetic acid disodium was dissolved in 54 mL buffered aqueous solution ([Na_2_HPO_4_] = [NaH_2_PO_4_] = 0.1 mol L^−1^), and then, 3.272 g Bi(NO_3_)_3_·5H_2_O was added to the solution, which was stirred at room temperature. Separately, 1.646 g NaVO_3_ was added to 27 mL buffered aqueous solution and sonicated at room temperature. Then, the two solutions were mixed and stirred at room temperature until a clear solution formed. A certain amount of sodium chloride was then dissolved in the solution. The pH of the solution was adjusted to 7 by addition of 3 M NaOH solution. The FTO substrate bearing the prefabricated BiVO_4_ seed layer was immersed in the reaction solution with the seed layer facing down and allowed to react at 80 °C for 6 h followed by heat treatment at 450 °C for 3 h.

### Material Characterization

The morphologies and microstructures of the samples were studied by field-emission scanning electron microscopy (FE-SEM, Nova NanoSEM NPE218) and transmission electron microscopy (TEM, JEM-2100F, JEOL, Japan). X-ray diffractometry (XRD, AXS-8 Advance, Bruker, Germany) was used to determine the crystal phases of the prepared samples. The UV–visible absorption spectra of the samples were recorded on a UV–Vis spectrophotometer (TU-1901, Beijing Purkinje General Instrument Co.).

### PEC Measurements

The PEC tests were performed using an electrochemical workstation (CHI 660c, CH Instruments Inc., USA). A 300-W xenon lamp (Beijing Perfectlight Technology Co., Ltd.) was used as a simulated-solar-light source, and all experiments were carried out under AM1.5 (light density 100 mW cm^−2^) solar-light illumination from the FTO side. Linear sweep voltammetry (LSV) analysis of the photoelectrodes was performed with a 0.1 M KH_2_PO_4_ (pH 7) electrolyte using a three-electrode system with platinum foil as the counter-electrode, a Ag/AgCl reference electrode, and the prepared photoelectrode as the working electrode. Incident photon-to-charge conversion efficiency (IPCE) was measured using a monochromator (Zolix, China), a 500-W xenon arc lamp, calibrated silicon photodetector, and a power meter. Intensity-modulated photocurrent spectroscopy (IMPS) was carried out using an electrochemical workstation (Zennium, effect-Elektrik, Germany) equipped with a controlled intensity-modulated spectrophotometry set-up (CIMPS, PP211) in a three-electrode configuration with the prepared film as the working electrode, a platinum foil as the counter-electrode, and a Ag/AgCl reference electrode with a 0.1 M KH_2_PO_4_ electrolyte at 1.23 V versus RHE. Modulated light in the frequency range 0.1–10 kHz was applied. The transient photocurrent was assessed using an electrochemical workstation (Zennium; effect-Elektrik, Germany) with the same three-electrode system as that used for the PEC measurements. The PEC degradation of methylene blue was conducted at pH 7 under vigorous stirring, AM1.5 irradiation, an electric bias of 0.6 V (vs. Ag/AgCl), and a 0.1 mol L^−1^ Na_2_SO_4_ supporting electrolyte. Hydrogen evolution was measured in a quartz device (LabSolar-IIIAG, Perfect Light, China), which included a gas-collection system and a reactor. This device was connected to a gas chromatographer (GC; GC-2010plus, Shimadzu, Japan). At certain time intervals during the test, a certain amount of gas in the gas-collection system was sent to the GC system to determine the amount of H_2_ produced. Mott–Schottky plots were obtained in the dark with an applied frequency of 1 kHz. Electrochemical impedance spectroscopy (EIS) was performed at 1.23 V versus RHE over a frequency range of 1 Hz–10 kHz under simulated-sunlight conditions.

## Results and Discussion

### Characterization of BiVO_4_ Films

**Fig. 1:**
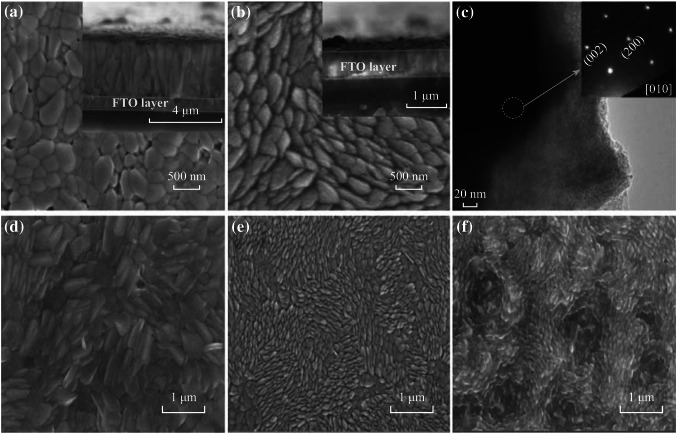
SEM images of the surface of BiVO_4_ photoanodes deposited at 80 °C for 6 h in the precursor solution with the addition of **a** no NaCl, **b, e** 5.85 g NaCl, **d** 2.93 g NaCl and **f** 11.7 g NaCl. **c** TEM image of the BiVO_4_ film prepared by adding 5.85 g NaCl in the precursor solution, the inset is the SAED pattern. The insets in **a** and **b** are the corresponding cross-sectional SEM images of BiVO_4_ photoanodes

Figure 1 shows the SEM images of the surface of BiVO_4_ photoanodes deposited at 80 °C for 6 h in precursor solutions with and without the addition of NaCl. When no NaCl is added, a compact nanocrystalline BiVO_4_ film is formed. Addition of NaCl markedly changes the morphology of the film (Fig. 1b) and decreases its thickness from 2.53 μm to 250 nm. This is because the occurrence of Cl^−^ inhibits the formation of BiVO_4_ by changing the composition of the growing unit (as has also been reported by Moon [29]) and Cl^−^ readily adsorbs on certain crystal planes, thus suppressing the growth of BiVO_4_ in those directions. As shown in Fig. 1d, when insufficient NaCl is added, crystal grain refinement starts to show and the lateral facet begins to be exposed, but when excess NaCl is added (Fig. 1f), the ordering of the grain is randomized and, more importantly, the growth of the grain on the substrate is slower, meaning that a film of effective thickness cannot be formed in a reasonable time. Therefore, the amount of NaCl must be accurately controlled. Figure S1a–d shows an energy dispersive spectroscopy (EDS) layered image and element maps for Bi, V, and O, revealing that no Cl is present on the BiVO_4_ surface. Figure 1c shows a TEM image with the corresponding selected area electron diffraction (SAED) pattern of BiVO_4_. The clear SAED pattern (indexed as the [010] zone) indicates that the BiVO_4_ is very crystalline. The SAED analyses suggest that the BiVO_4_ film preferentially grows along the (040) facets. These findings are confirmed by the XRD results discussed later.

**Fig. 2:**
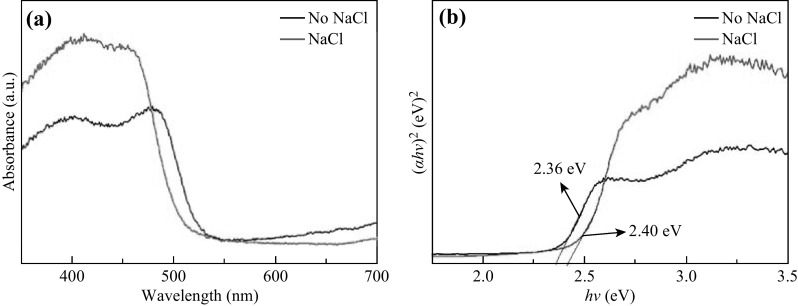
**a** Absorption spectra of BiVO_4_ photoanodes deposited at 80 °C for 6 h in the precursor solution. **b** The plot of (*αhv*)^2^ versus energy (*hv*) for the band-gap energy of BiVO_4_ films

The UV–Vis absorption spectra of BiVO_4_ photoanodes deposited at 80 °C for 6 h are shown in Fig. 2. They clearly indicate that the addition of NaCl to the precursor solution increases the absorption intensities of the BiVO_4_ films. The estimated *E*g values of the BiVO_4_ films prepared with and without NaCl in the precursor solution are 2.40 and 2.36 eV, respectively, as shown in Fig. 2b, and these are similar values to those reported by other authors [31–33]. The band-gap energy of a semiconductor material depends upon many physical and physicochemical parameters, such as size or dimensions, crystallinity, amount of vacancies, defects, and doping [34, 35]. Therefore, materials prepared by different methods may present different band gaps.

**Fig. 3:**
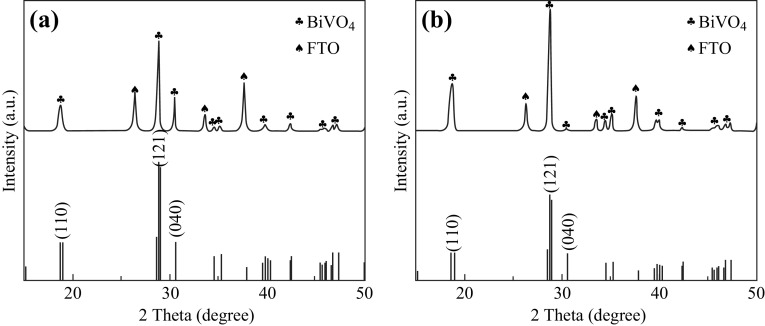
XRD patterns of the BiVO_4_ films prepared **a** without NaCl and **b** with NaCl

**Fig. 4:**
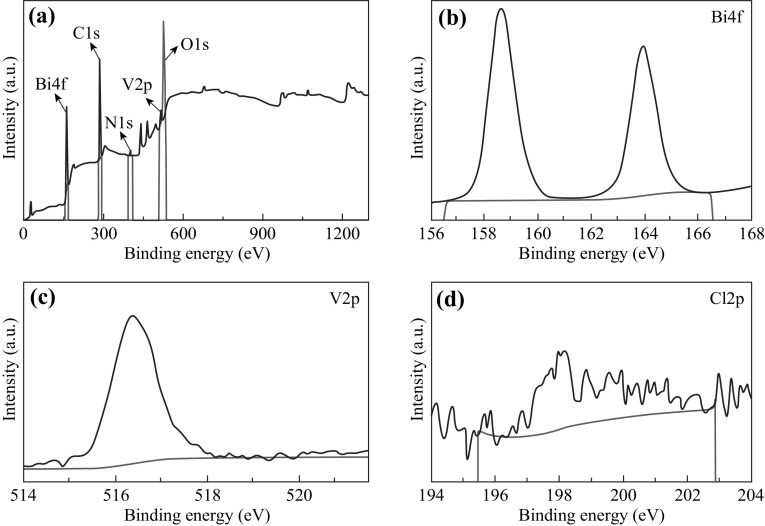
**a** X-ray photoelectron spectra of BiVO_4_ films prepared with NaCl, **b** Bi 4f spectra, **c** V 2p spectra, and **d** Cl 2p spectra

Figure 3 shows XRD patterns of BiVO_4_ films prepared with and without the addition of NaCl to the precursor solution. It can be seen that the BiVO_4_ films have a single monoclinic scheelite structure. For the film prepared with NaCl, the diffraction peak at 30.8° corresponding to the (040) plane is almost absent, indicating that the [040] direction is parallel to the FTO substrate. Conversely, the film prepared without NaCl exhibits a sharp diffraction peak at 30.8° (Fig. 3a). According to the SEM images, the addition of NaCl results in an increase of the (040) plane, which improves PEC activity owing to the existence of built-in electric fields between different facets [36]. The X-ray photoelectron spectra of the BiVO_4_ film prepared with NaCl are shown in Fig. 4 and indicates the presence of Bi, V, and O. Again, no Cl is detected, similarly to the spectra of the BiVO_4_ film prepared without NaCl (Fig. S2).

### Photoelectrochemical Properties of BiVO_4_ Films

**Fig. 5:**
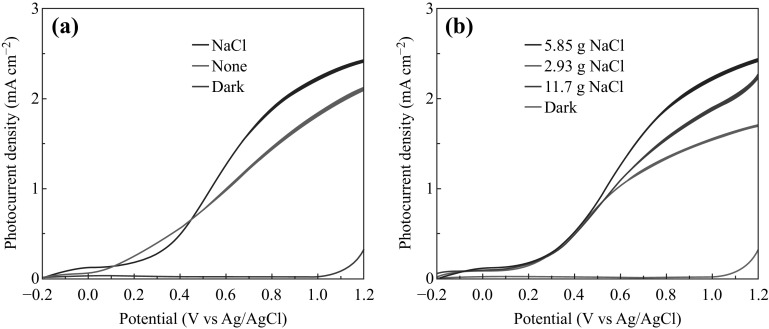
*I*–*V* curves for PEC water oxidation of the BiVO_4_ films measured in 0.1 M KH_2_PO_4_ electrolyte under AM1.5G illumination

**Fig. 6:**
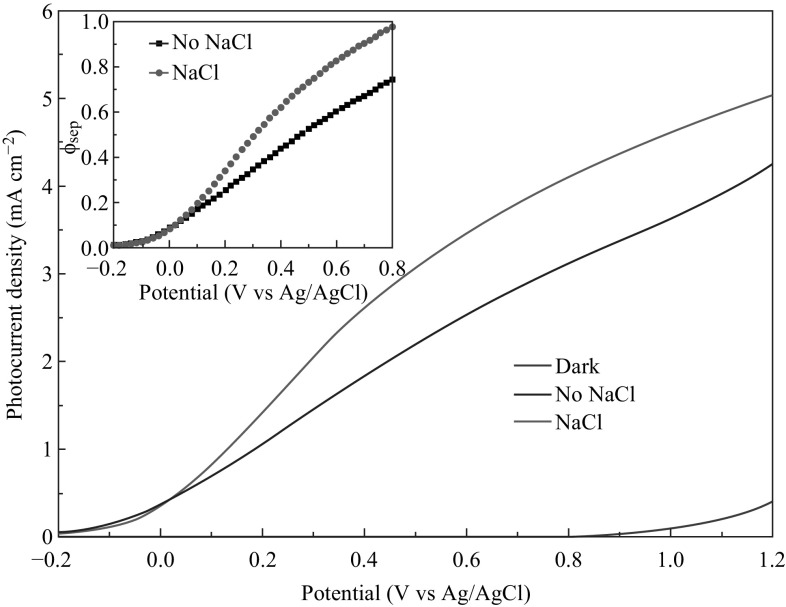
*I*–*V* curves of BiVO_4_ electrodes measured in a 0.1 M phosphate buffer (pH 7) containing 0.1 M Na_2_SO_3_ as hole scavenger under AM1.5G, 100 mW cm^−2^ illumination (scan rate, 50 mV s^−1^). The inset is *ϕ*_sep_ calculated from the *I*–*V* curves after dark current is subtracted

The *I*–*V* curves for water oxidation over the BiVO_4_ films deposited at 80 °C for 6 h are shown in Fig. 5. The BiVO_4_ film prepared with NaCl achieves a photocurrent of 1.26 mA cm^−2^ at 0.6 V versus Ag/AgCl, which is one of the highest photocurrent densities reported to date for bare BiVO_4_ photoanodes (see Table S1 for a comparison with similar BiVO_4_ photoanodes), while the BiVO_4_ film prepared without NaCl exhibits a photocurrent of only 0.88 mA cm^−2^. It can be seen from Fig. 5b that with increasing NaCl content, the photocurrent densities of the photoanodes first increase and then decrease. The exposure and order degrees of the (040) facets affect the PEC activities of the BiVO_4_ photoanodes. In order to investigate the reasons for the increase in photocurrent, the PEC properties of the photoanodes were assessed in the presence of 0.1 M sodium sulfite, which serves as a hole scavenger. Typical *I*–*V* curves for sulfite oxidation over the BiVO_4_ films are shown in Fig. 6.

The photocurrent of water splitting ($J_{\text{photocurrent}}^{{{\text{H}}_{2} {\text{O}}}}$ ) can be calculated according to Eq. 1,

1 $$ J^{{{\text{H}}_{2} {\text{O}}}}_{\text{photocurrent}} = J_{\text{absorbed}} \times P_{\text{charge\,separation}} \times P_{\text{charge\,injection}} $$

where *P*_charge separation_ is the charge-separation yield of the photogenerated carriers, *P*_charge injection_ is the charge-injection yield to the electrolyte, and *J*_absorbed_ is the rate of photon absorption expressed as current density. The photocurrent obtained for sulfite oxidation is a product of *J*_absorbed_ and *P*_charge separation_ alone, assuming the charge-injection yield is 100% (*P*_charge injection_ = 1) in the presence of a hole scavenger (Na_2_SO_3_) in the electrolyte. Therefore, *P*_charge separation_ can be obtained by dividing $J_{\text{photocurrent}}^{{{\text{Na}}_{2} {\text{SO}}_{3} }}$ by *J*_absorbed_ (Fig. 6, inset). The results show that BiVO_4_ films prepared with and without NaCl achieve *P*_charge separation_ values of 0.82 and 0.60 at 0.6 V versus Ag/AgCl, respectively. The significant improvement upon addition of NaCl can be ascribed to the exposure of (040) facets, which causes charge-transfer anisotropy due to the existence of built-in electric fields between different facets of BiVO_4_ [36].

The charge-injection efficiency of photoelectrodes can be calculated according to Eq. 2 using the data in Figs. 5 and 6.

2 $$ P_{\text{charge\,injection}} = J^{{{\text{H}}_{ 2} {\text{O}}}}_{\text{photocurrent}} /J^{{{\text{Na}}_{2} {\text{SO}}_{3} }}_{\text{photocurrent}} $$

Figure S3 shows that the charge-injection efficiencies for BiVO_4_ films prepared with and without NaCl at 0.6 V versus Ag/AgCl are 0.367 and 0.349, respectively. In general, the charge-injection efficiencies are almost the same for the two BiVO_4_ films, and thus, the main reason for the difference in the photocurrent is the imparity of their separation efficiencies. And it is easy to understand because no co-catalyst was modified on the surface; thus, the majority of the surface-reaching holes were lost to surface recombination because of the poor catalytic nature of the BiVO_4_ surface for water oxidation.

**Fig. 7:**
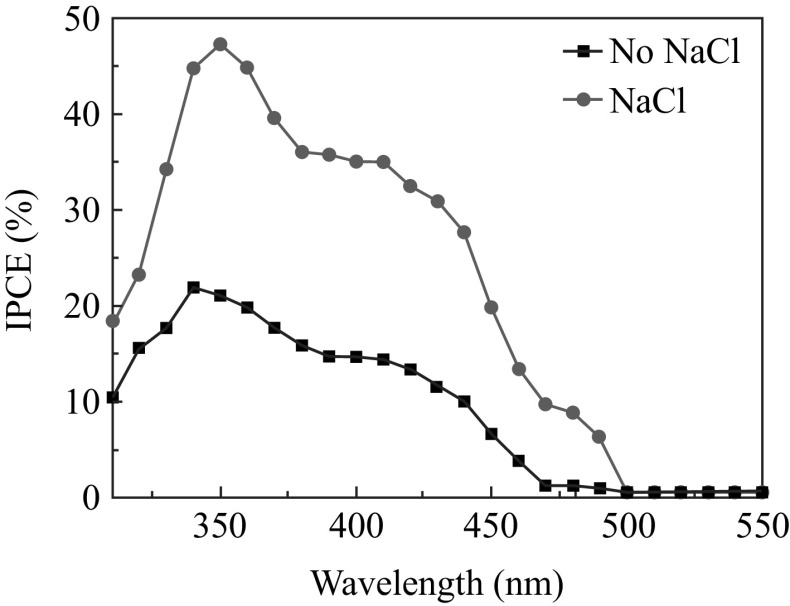
IPCE plots of the BiVO_4_ films measured at 1.23 V versus RHE in 0.1 M KH_2_PO_4_ electrolyte

The IPCE values of the prepared samples at 1.23 V versus RHE can be obtained using Eq. 3 [37, 38],

3 $$ {\text{IPCE }}\left( \% \right) = \left( {1240 \, \times I} \right)/\left( {\lambda \, \times J_{\text{light}} } \right) $$

where *I* is the photocurrent density measured under monochromatic light, *λ* is the incident light wavelength, and *J*_light_ is the measured irradiance. The IPCE values at 400 nm for BiVO_4_ photoanodes prepared with and without NaCl are approximately 35% and 14.6%, respectively (Fig. 7). Based on IPCE (%) = charge-separation efficiency (*P*_sep_) × charge-transport efficiency (*P*_trans_) × interfacial charge-transfer efficiency (*P*_inter_) at the interfacial solid–liquid junction, the IPCE characteristics are consistent with the fact that the addition of NaCl leads to higher solar-light absorption and charge-separation efficiency, considering that *P*_inter_ is regarded for use of a co-catalyst.

Mott–Schottky analysis was performed in the dark using a 0.1 M KH_2_PO_4_ electrolyte (Fig. S4). As expected for an n-type semiconductor, both samples show positive slopes, and the flat-band potentials of the BiVO_4_ photoanodes prepared with and without NaCl are −0.036 and 0.177 V versus RHE, respectively. Equation 4 was used to calculate the carrier density of each sample.

4 $$ N_{d} = \, \left( {2/e\varepsilon \varepsilon_{0} } \right)\left[ {d\left( {1/C^{2} } \right)/dV} \right]^{ - 1} $$

where *e* is the electron charge, *ε* is the relative dielectric constant of BiVO_4_, *ε*_0_ is the permittivity of a vacuum (8.85 × 10^−12^ F m^−1^), and *V* is the applied bias at the electrode. As shown in Fig. S4, the BiVO_4_ film prepared with NaCl shows a decreased slope, which indicates the rapid increase in *N*_d_. According to the equation, the calculated carrier densities of BiVO_4_ films prepared with and without NaCl are 5.93 × 10^19^ and 2.34 × 10^19^ cm^−3^, respectively.

EIS was performed at 1.23 V versus RHE under simulated-sunlight irradiation. As shown in Fig. S5, the BiVO_4_ photoanode with exposed (040) facets exhibits a smaller impedance arc diameter than that of the BiVO_4_ film prepared without NaCl, indicating that charge transfer across the electrode/electrolyte interface is more favorable for the BiVO_4_ photoanode with exposed (040) facets. The higher charge-transfer ability at the interface diminishes charge recombination and induces the transport of electrons through the films, and this is supported by the higher carrier density of the BiVO_4_ photoanode with exposed (040) facets as revealed by *M*–*S* analysis, shown in Fig. S4.

**Fig. 8:**
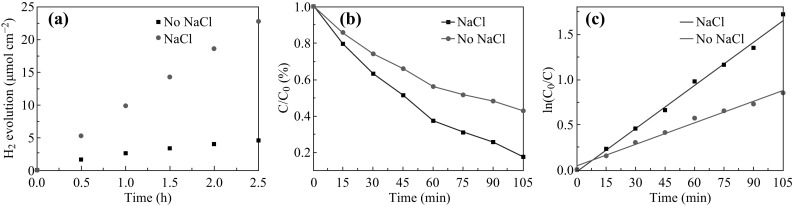
**a** The water-splitting performance of BiVO_4_ photoanodes tested at 0.6 V versus Ag/AgCl in 0.1 M KH_2_PO_4_ electrolyte under AM1.5 illumination. **b** Degradation of methylene blue by PEC system using BiVO_4_ photoanodes prepared with and without NaCl, and **c** the corresponding kinetics curves

The PEC water-splitting performances of the BiVO_4_ photoanodes were assessed in 0.1 M KH_2_PO_4_ electrolyte (pH 7) at 0.6 V versus Ag/AgCl under AM1.5 illumination. As shown in Fig. 8a, during the 150-min test, the H_2_ evolution rates over BiVO_4_ photoanodes prepared with and without NaCl are 22.77 and 4.55 μmol cm^−2^, respectively, which demonstrates that the exposure of (040) facets dramatically improves water-splitting activity. The faradaic efficiencies (*η)* of the BiVO_4_ photoanodes were calculated according to Eq. 5:

5 $$ \eta = \, \alpha Fn/Q_{J} \times 100\% $$

where *α* = 2 (H_2_) or 4 (O_2_), *F* = 96,485 C mol^−1^, *n* is the yield of H_2_ or O_2_, and *Q*_*J*_ is the amount of electricity through the external circuit. As shown in Fig. S6, the faradaic efficiency for O_2_ is 24% for the BiVO_4_ photoanode with (040) facets exposed and 2.5% for the BiVO_4_ photoanode prepared without NaCl. In contrast, the faradaic efficiencies for H_2_ for the two BiVO_4_ photoanodes are essentially the same (> 96%), indicating that the Pt cathode represents a stable and efficient electrode for the hydrogen evolution reaction (HER).

The long-term stability of the BiVO_4_ photoanodes during water oxidation was also assessed (shown in Fig. S7). Compared with the BiVO_4_ photoanode prepared without NaCl, the photoanode with exposed (040) facets shows greatly enhanced stability. The photocurrent density of the BiVO_4_ photoanode with exposed (040) facets only decreases by 14% over 2 h, while that of the BiVO_4_ film prepared without NaCl almost reaches zero after 2 h. As can be seen from Fig. S8, the morphology of the BiVO_4_ photoanode with exposed (040) facets is almost unchanged, while obvious corrosion occurs on the surface of the BiVO_4_ photoanode prepared without NaCl. This result confirms the enhanced stability when more (040) facets are exposed.

The PEC activities of the BiVO_4_ films were also investigated experimentally by organic compound degradation [39]. The PEC (at a bias potential of 0.6 V) degradation of methylene blue (MB) in neutral aqueous solution was performed under the given conditions. As shown in Fig. 8b, the BiVO_4_ photoanode prepared without NaCl effected only 57% removal over 105 min, while the BiVO_4_ photoanode prepared with NaCl effected 82% removal over 105 min. The rate constant for PEC degradation with the BiVO_4_ photoanode prepared using NaCl (0.958 h^−1^) is twice that for the BiVO_4_ photoanode prepared without NaCl (0.478 h^−1^), which can be ascribed to the exposure of the (040) facets.

### IMPS and Transient Photocurrent Measurements

IMPS is considered to be a powerful method for obtaining information about the photogenerated-carrier-transport properties and carrier recombination of a semiconductor. In this case, the average transit time required by photogenerated electrons to reach the back contact was used as an index for the recombination probability of photogenerated electrons and holes. The transit time *τ*_d_ can be calculated according to the formula *τ*_*d*_ = (2π*f*_min_)^−1^, where *f*_min_ is the characteristic frequency at which the minimum value occurs in the IMPS plot [40].

**Fig. 9:**
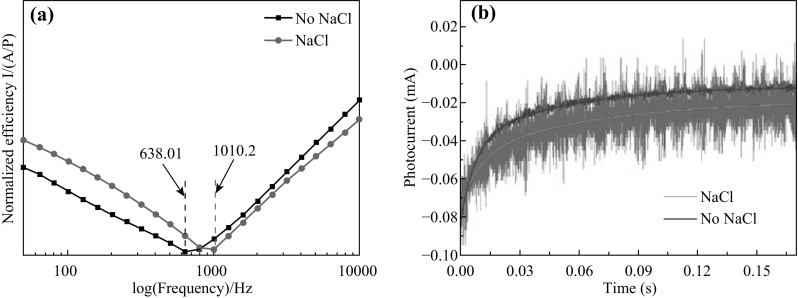
**a** The IMPS plots of the normalized efficiency versus log (frequency). **b** Transient photocurrent for the BiVO_4_ films prepared with and without NaCl. Bias illumination: white light, 80 mW cm^−2^

As shown in Fig. 9a, the *f*min values for BiVO_4_ films prepared with and without NaCl are 1010.2 and 638.01 Hz, respectively. Thus, we obtain *τ*_*d*_ values of 0.157 and 0.250 ms, respectively. *τ*_*d*_ for the BiVO_4_ film prepared without NaCl is almost two times that of the BiVO_4_ film prepared with NaCl, demonstrating that the photogenerated charges more easily reach the back contact in the BiVO_4_ film prepared by adding NaCl to the precursor solution.

To investigate how the exposure of (040) facets improves the PEC performances of BiVO_4_ photoanodes, transient photocurrent (TP) measurement was also performed to study the dynamics of the photoinduced charge carriers formed in the BiVO_4_ photoanodes [41–43] (Fig. 9b). The relaxation of the TP signals can be characterized in terms of two time constants (*τ*_1_ and *τ*_2_) with corresponding probabilities (φ_1_ and φ_2_, see SI for detailed information), which reveal the lifetimes of the trapped holes. The average decay times (*τ*) are 15.88 and 7.15 ms at 1.23 *V*_RHE_ for the BiVO_4_ photoanodes prepared with and without NaCl, respectively, which indicates that the exposure of (040) facets suppresses charge recombination at the interfacial solid–liquid junction.

## Conclusion

A facile method for the preparation of highly efficient BiVO_4_ photoanodes with exposed (040) facets is demonstrated in this study. By adding NaCl to the precursor solution, more (040) crystal planes of the BiVO_4_ photoanode are exposed, leading to higher light adsorption and charge-separation efficiency. Thus, one of the highest photocurrent densities reported for such unmodified photoanodes has been achieved. The BiVO_4_ photoanode prepared by adding NaCl demonstrated a photocurrent density of 1.26 mA cm^−2^ at 1.23 V versus RHE in a 0.1 M KH_2_PO_4_ (pH 7) electrolyte under simulated AM1.5G solar light and an IPCE above 35% at 400 nm. The BiVO_4_ photoanode also demonstrated excellent PEC degradation efficiency, water-splitting performance, and stability. This study provides a promising strategy for the preparation of highly efficient BiVO_4_ photoanodes for PEC applications.

## Electronic Supplementary Material

Below is the link to the electronic supplementary material.

Supplementary material 1 (PDF 1242 kb)

## References

[1] Zhang P, Zhang J, Gong J (2014). Tantalum-based semiconductors for solar water splitting. Chem. Soc. Rev..

[2] Osterloh FE (2013). Inorganic nanostructures for photoelectrochemical and photocatalytic water splitting. Chem. Soc. Rev..

[3] Kudo A, Miseki Y (2009). Heterogeneous photocatalyst materials for water splitting. Chem. Soc. Rev..

[4] Graetz J (2009). New approaches to hydrogen storage. Chem. Soc. Rev..

[5] Jia Q, Iwashina K, Kudo A (2012). Facile fabrication of an efficient BiVO_4_ thin film electrode for water splitting under visible light irradiation. Proc. Natl. Acad. Sci..

[6] Xie M, Fu X, Jing L, Luan P, Feng Y, Fu H (2014). Long-lived, visible-light-excited charge carriers of TiO_2_/BiVO_4_ nanocomposites and their unexpected photoactivity for water splitting. Adv. Energy Mater..

[7] Long M, Jiang J, Li Y, Cao R, Zhang L, Cai W (2011). Effect of gold nanoparticles on the photocatalytic and photoelectrochemical performance of Au modified BiVO_4_. Nano-Micro Lett..

[8] Zhang X, Quan X, Chen S, Zhang Y (2010). Effect of Si doping on photoelectrocatalytic decomposition of phenol of BiVO_4_ film under visible light. J. Hazard. Mater..

[9] Li M, Zhao L, Guo L (2010). Preparation and photoelectrochemical study of BiVO_4_ thin films deposited by ultrasonic spray pyrolysis. Int. J. Hydrogen Energy.

[10] Yao W, Iwai H, Ye J (2008). Effects of molybdenum substitution on the photocatalytic behavior of BiVO_4_. Dalton Trans..

[11] Wang G, Ling Y, Lu X, Qian F, Tong Y, Zhang JZ, Lordi V, Rocha Leao C, Li Y (2013). Computational and photoelectrochemical study of hydrogenated bismuth vanadate. J. Phys. Chem. C.

[12] Zhang Y, Zhang X, Wang D, Wan F, Liu Y (2017). Protecting hydrogenation-generated oxygen vacancies in BiVO_4_ photoanode for enhanced water oxidation with conformal ultrathin amorphous TiO_2_ layer. Appl. Sur. Sci..

[13] Cooper JK, Scott SB, Ling Y, Yang J, Hao S (2016). Role of hydrogen in defining the n-type character of BiVO_4_ photoanodes. Chem. Mater..

[14] Zhong DK, Choi S, Gamelin DR (2011). Near-complete suppression of surface recombination in solar photoelectrolysis by “Co-Pi” catalyst-modified W:BiVO_4_. J. Am. Chem. Soc..

[15] Abdi FF, van de Krol R (2012). Nature and light dependence of bulk recombination in Co-Pi catalyzed BiVO_4_ photoanodes. J. Phys. Chem. C.

[16] Cai P, Zhou S, Ma D, Liu S, Chen W, Huang S (2015). Fe_2_O_3_-modified porous BiVO_4_ nanoplates with enhanced photocatalytic activity. Nano-Micro Lett..

[17] Ho-Kimura SJA, Moniz AD, Handoko J (2014). Tang, Enhanced photoelectrochemical water splitting by nanostructured BiVO_4_-TiO_2_ composite electrodes. J. Mater. Chem. A.

[18] Fu X, Xie M, Luan P, Jing L (2014). Effective visible-excited charge separation in silicate-bridged ZnO/BiVO_4_ nanocomposite and its contribution to enhanced photocatalytic activity. ACS Appl. Mater. Interfaces.

[19] Moniz SJA, Zhu J, Tang J (2014). 1D Co-Pi modified BiVO_4_/ZnO junction cascade for efficient photoelectrochemical water cleavage. Adv. Energy Mater..

[20] Zhang L, Reisner E, Baumberg JJ (2014). Al-doped ZnO inverse opal networks as efficient electron collectors in BiVO_4_ photoanodes for solar water oxidation. Energy Environ. Sci..

[21] Kim JH, Magesh G, Kang HJ, Banu M, Kim JH, Lee J, Lee JS (2015). Carbonate-coordinated cobalt co-catalyzed BiVO_4_/WO_3_ composite photoanode tailored for CO_2_ reduction to fuels. Nano Energy.

[22] Wang R, Bai J, Li Y, Zeng Q, Li J, Zhou B (2017). BiVO_4_/TiO_2_(N_2_) nanotubes heterojunction photoanode for highly efficient photoelectrocatalytic applications. Nano-Micro Lett..

[23] Tan G, Zhang L, Ren H, Huang J, Yang W, Xia A (2014). Microwave hydrothermal synthesis of N-doped BiVO_4_ nanoplates with exposed (040) facets and enhanced visible-light photocatalytic properties. Ceram. Int..

[24] Li H, Sun Y, Cai B, Gan S, Han D, Niu L, Wu T (2015). Hierarchically Z-scheme photocatalyst of Ag@AgCl decorated on BiVO_4_ (040) with enhancing photoelectrochemical and photocatalytic performance. Appl. Catal. B Environ..

[25] Li G, Nie X, Chen J, Wong PK, An T, Yamashita H, Zhao H (2016). Enhanced simultaneous PEC eradication of bacteria and antibiotics by facilely fabricated high-activity 001 facets TiO_2_ mounted onto TiO_2_ nanotubular photoanode. Water Res..

[26] Zeng Q, Li J, Bai J, Li X, Xia L, Zhou B (2017). Preparation of vertically aligned WO_3_ nanoplate array films based on peroxotungstate reduction reaction and their excellent photoelectrocatalytic performance. Appl. Catal. B Environ..

[27] Hosono E, Fujihara S, Kakiuchi K, Imai H (2004). Growth of submicrometer-scale rectangular parallelepiped rutile TiO_2_ films in aqueous TiCl_3_ solutions under hydrothermal conditions. J. Am. Chem. Soc..

[28] Wang Y, Guo M, Zhang M, Wang X (2010). Hydrothermal preparation and photoelectrochemical performance of size-controlled SnO_2_ nanorod arrays. CrystEngComm.

[29] Lee H, Kim A, Kwon H, Yang W, Oh Y, Lee D, Moon J (2016). Retarding crystallization during facile single coating of NaCl-incorporated precursor solution for efficient large-area uniform perovskite solar cells. ACS Appl. Mater. Interfaces.

[30] Kim CW, Son YS, Kang MJ, Kim DY, Kang YS (2016). (040)-Crystal facet engineering of BiVO_4_ plate photoanodes for solar fuel production. Adv. Energy Mater..

[31] Luo W, Wang Z, Wan L, Li Z, Yu T, Zou Z (2010). Synthesis, growth mechanism and photoelectrochemical properties of BiVO_4_ microcrystal electrodes. J. Phys. D Appl. Phys..

[32] Yang L, Xiong Y, Dong H, Peng H, Zhang Y, Xiao P (2017). Enhanced charge separation and oxidation kinetics of BiVO_4_ photoanode by double layer structure. J. Power Sources.

[33] Wu Q, Bao S, Tian B, Xiao Y, Zhang J (2016). Double-diffusion-based synthesis of BiVO_4_ mesoporous single crystals with enhanced photocatalytic activity for oxygen evolution. Chem. Commun..

[34] Chatten R, Chadwick AV, Rougier A, Lindan PJD (2005). The oxygen vacancy in crystal phases of WO_3_. J. Phys. Chem. B.

[35] Ahmadi M, Sahoo S, Younesi R, Gaur APS, Katiyar RS (2014). M.J-F Guinel, WO_3_ nano-ribbons: their phase transformation from tungstite (WO_3_·H_2_O) to tungsten oxide (WO_3_). J. Mater. Sci..

[36] Zhu J, Fan F, Chen R, An H, Feng Z, Li C (2015). Direct imaging of highly anisotropic photogenerated charge separations on different facets of a single BiVO_4_ photocatalyst. Angew. Chem. Int. Ed..

[37] Ye K, Chai Z, Gu J, Yu X, Zhao C, Zhang Y, Mai W (2015). BiOI-BiVO_4_ photoanodes with significantly improved solar water splitting capability: p-n junction to expand solar adsorption range and facilitate charge carrier dynamics. Nano Energy.

[38] Kim ES, Kang HJ, Magesh G, Kim JY, Jang J, Lee JS (2014). Improved photoelectrochemical activity of CaFe_2_O_4_/BiVO_4_ heterojunction photoanode by reduced surface recombination in solar water oxidation. ACS Appl. Mater. Interfaces.

[39] Dong Y, Li J, Li X, Zhou B (2016). The promotion effect of low-molecular hydroxyl compounds on the nano-photoelectrocatalytic degradation of fulvic acid and mechanism. Nano-Micro Lett..

[40] Su J, Guo L, Bao N, Grimes CA (2011). Nanostructured WO_3_/BiVO_4_ heterojunction films for efficient photoelectrochemical water splitting. Nano Lett..

[41] Pesci FM, Cowan AJ, Alexander BD, Durrant JR, Klug DR (2011). Charge carrier dynamics on mesoporous WO_3_ during water splitting. J. Phys. Chem. Lett..

[42] Wang Y, Wang H-Y, Yu M, Fu L-M, Qin Y, Zhang J-P, Ai X-C (2015). Trap-limited charge recombination in intrinsic perovskite film and meso-superstructured perovskite solar cells and the passivation effect of the hole-transport material on trap states. Phys. Chem. Chem. Phys..

[43] Yoshihara T, Tamaki Y, Furube A, Murai M, Hara K, Katoh R (2007). Effect of pH on absorption spectra of photogenerated holes in nanocrystalline TiO_2_ films. Chem. Phys. Lett..

