# Supplementary material for: BiVO4 Photoanode with Exposed (040) Facets for Enhanced Photoelectrochemical Performance
Source: Nanomicro Lett. 2017 Oct 31;10(1):11. doi: 10.1007/s40820-017-0163-3 (PMC6199060; doi:10.1007/s40820-017-0163-3)
Supplement: Supplementary file 1 — Supplementary material 1 (PDF 1242 kb) [file 40820_2017_163_MOESM1_ESM.pdf]

Supporting Information for

# $BiVO_4$ Photoanode with Exposed (040) Facets for Enhanced Photoelectrochemical Performance

Ligang Xia[1], Jinhua Li[1], Jing Bai[1,*], Linsen Li[1], Shuai Chen[1], Baoxue Zhou[1,2,*]

[1]School of Environmental Science and Engineering, Shanghai Jiao Tong University No. 800 Dongchuan Rd, Shanghai 200240, People's Republic of China
[2]Key Laboratory of Thin Film and Microfabrication Technology, Ministry of Education, Shanghai 200240, People's Republic of China

∗Corresponding authors. E-mail: zhoubaoxue@sjtu.edu.cn; bai_jing@sjtu.edu.cn
Tel: +86-21-5474 7351; Fax: +86-21-5474 7351

## 1 Figures and Table

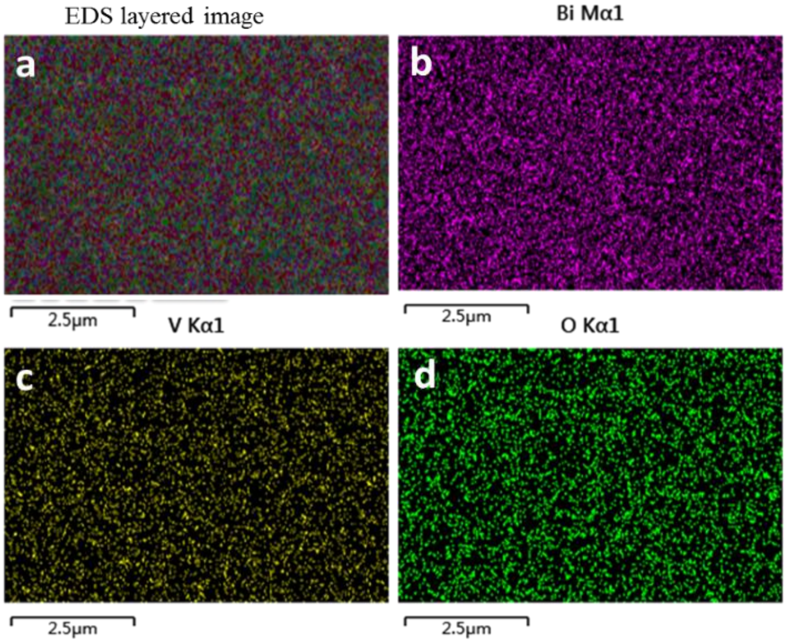


**Fig. S1** **a** EDS layered image and the corresponding STEM-EDS elemental mapping images of **b** Bi, **c** V, and **d** O

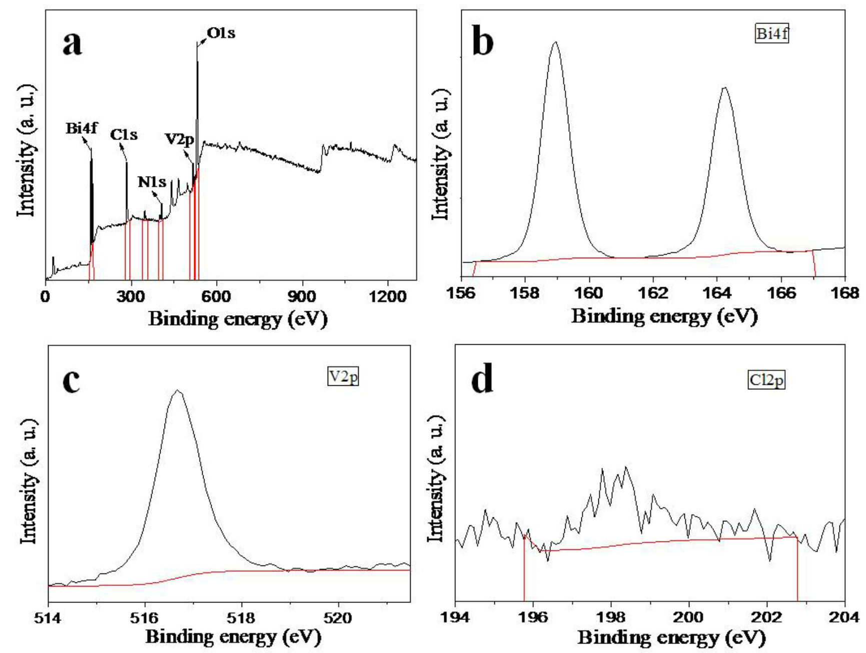


**Fig. S2** **a** XPS survey data and **b** Bi 4f, **c** V 2p, **d** Cl 2p spectra of $BiVO_4$ films prepared without NaCl

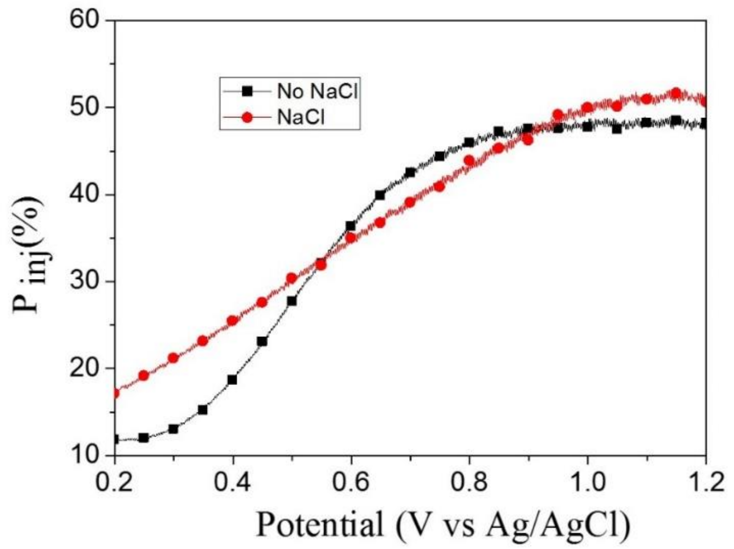


**Fig. S3** Charge injection efficiency versus potential curves of $BiVO_4$ films prepared with and without NaCl

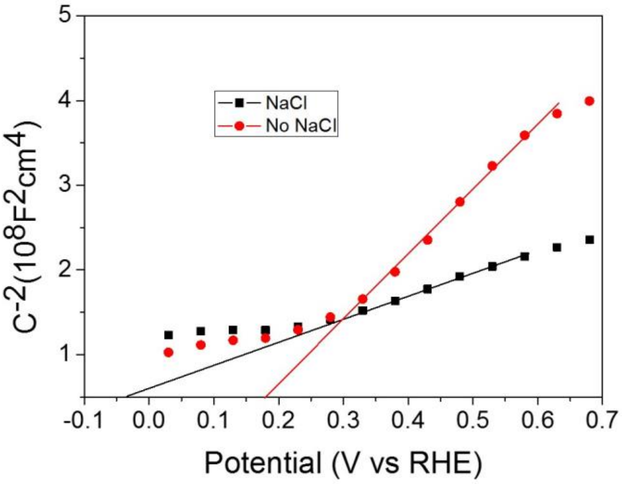


**Fig. S4** Mott–Schottky plots for $BiVO_4$ photoanodes prepared with and without NaCl

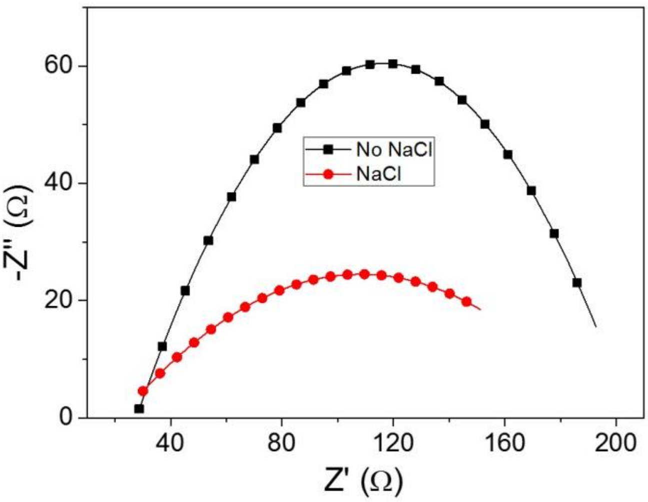


**Fig. S5** Nyquist plots of the $BiVO_4$ photoanodes prepared with and without NaCl

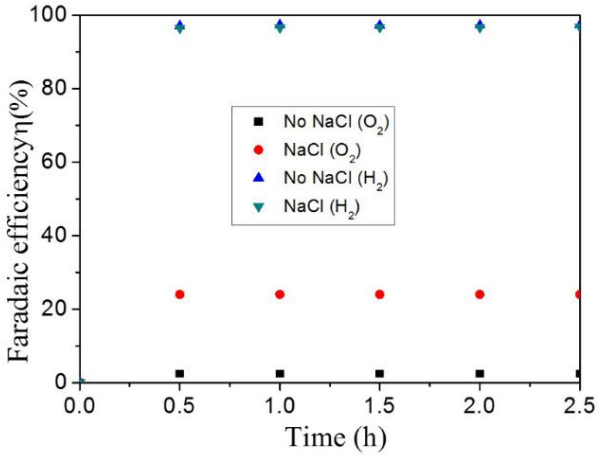


**Fig. S6** Faradaic efficiency of the $BiVO_4$ photoanodes for water oxidation

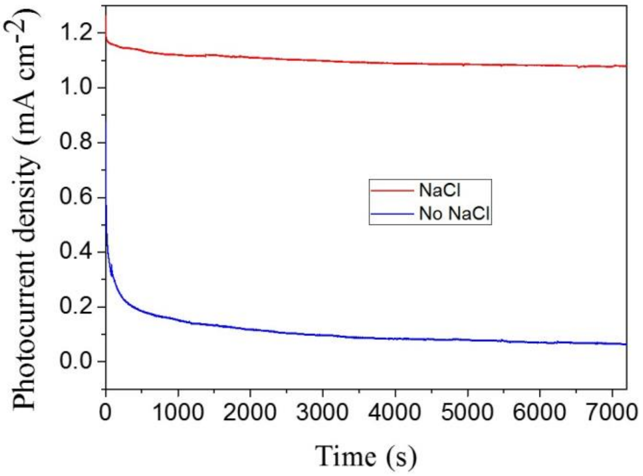


**Fig. S7** *I-t* curves of $BiVO_4$ photoanodes prepared with and without NaCl at 1.23 V vs RHE under AM 1.5G illumination

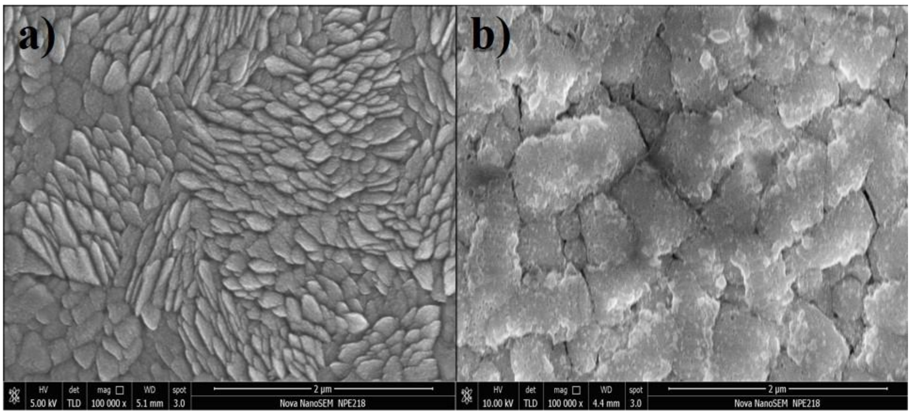


**Fig. S8** SEM images of $BiVO_4$ photoanodes prepared with **a** and without **b** NaCl after water splitting

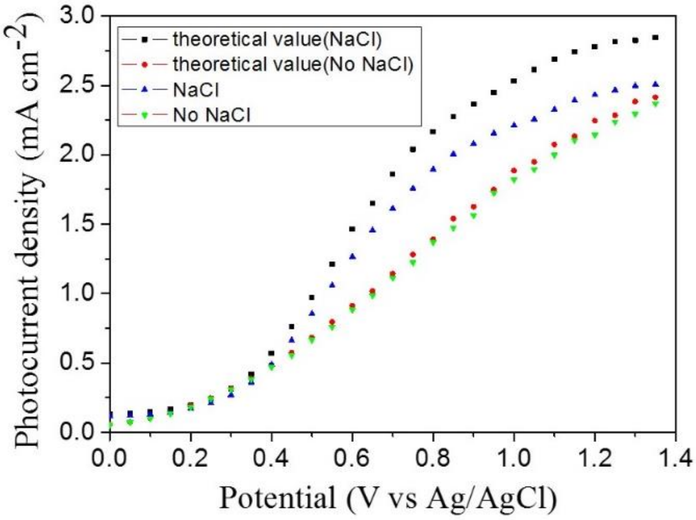


**Fig. S9** The comparison of the theoretical and measured photocurrent density

The theoretical current density was slightly higher than the measured one (shown in Figure. S9), which may be ascribed to the limitations of the equation (Photocurrent density=theoretical maximum photocurrent value × light absorption efficiency × charge transfer efficiency × charge transport efficiency × faradaic efficiency) especially the theoretical maximum photocurrent density achievable assuming 100% IPCE for photons with energy $\geq E_g$ and calculated $J_{abs}$ assuming 100% APCE. The detailed calculation process can be refered to the literature [5].

**Table S1** Comparison of photoactivities with similar $BiVO_4$ photoanodes

| Bare $BiVO_4$ | Photocurrent density (mA $cm^{-2}$@1.23 V *vs* RHE) | IPCE@400 nm 1.23 V *vs* RHE | Reference |
|---|---|---|---|
| | <0.5 (200 mW $cm^{-2}$) | / | [1] |
| | <0.1 (158 mW $cm^{-2}$) | / | [2] |
| | 0.94 (100 mW $cm^{-2}$) | 42.1% @1.6 V *vs* RHE | [3] |
| | 0.25 (100 mW $cm^{-2}$) | / | [4] |
| | 1.26 (100 mW $cm^{-2}$) | >35% | This work |

## 2 Transient Photocurrent Analyses

The decay curves were fitted to a second-order exponential function [6-8],

$$y = y_0 + A_1 e^{-x/\tau_1} + A_2 e^{-x/\tau_2}$$

where $\tau_1$ and $\tau_2$ are the time constants and $A_1$ and $A_2$ are the probability constants. The percentage of $\tau_1$ ($\varphi_1$) was calculated as Eq. S1

$$\varphi_1 = \frac{A_1}{A_1 + A_2} \times 100\% \qquad \text{(S1)}$$

and the percentage of $\tau_2$ ($\varphi_2$) was calculated as Eq. S2

$$\varphi_2 = \frac{A_2}{A_1 + A_2} \times 100\% \qquad \text{(S2)}$$

The average decay time ($\tau$) was calculated as Eq. S3

$$\tau = \tau_1 \varphi_1 + \tau_2 \varphi_2 \qquad \text{(S3)}$$

## References


[1] M. Zhou, G. Xi, J. Ye, Synthesis of bismuth vanadate nanoplates with exposed {001} facets and enhanced visible-light photocatalytic properties. Chem. Commun. **46**(11), 1893-1895 (2010). doi:10.1039/b923435g

[2] M. Zhou, S. Zhang, Y. Sun, C. Wu, M. Wang, Y. Xie, C-oriented and {010} facets exposed $BiVO_4$ nanowall films: template-free fabrication and their enhanced photoelectrochemical properties. Chem. Asian J. **5**(12), 2515-2523 (2010). doi:10.1002/asia.201000452

[3] C.W. Kim, Y.S. Son, M.J. Kang, D.Y. Kim, Y.S. Kang, (040)-Crystal facet

engineering of $BiVO_4$ plate photoanodes for solar fuel production. Adv. Energy Mater. **6** (4), 1501754 (2016). doi:10.1002/aenm.201501754
[4] S. Wang, P. Chen, J. Yun, Y. Hu, L. Wang, Electrochemically-treated $BiVO_4$ photoanode for efficient photoelectrochemical water splitting. Angew. Chem. Int. Ed. **56**(29), 8500-8504 (2017). doi:10.1002/anie.201703491
[5] T.W. Kim, K.-S. Choi, Nanoporous $BiVO_4$ photoanodes with dual-layer oxygen evolution catalysts for solar water splitting. Science **343**(6174), 990-994 (2014). doi:10.1126/science.1246913
[6] F.M. Pesci, A.J. Cowan, B.D. Alexander, J.R. Durrant, D.R. Klug, Charge Carrier Dynamics on Mesoporous $WO_3$ during Water Splitting. J. Phys. Chem. Lett. **2**(15), 1900-1903 (2011). doi:10.1021/jz200839n
[7] Y. Wang, H.-Y. Wang, M. Yu, L.-M. Fu, Y. Qin, J.-P. Zhang, X.-C. Ai, Trap-limited charge recombination in intrinsic perovskite film and meso-superstructured perovskite solar cells and the passivation effect of the hole-transport material on trap states. Phys. Chem. Chem. Phys. **17**(44), 29501–29506 (2015). doi:10.1039/C5CP04360C
[8] T. Yoshihara, Y. Tamaki, A. Furube, M. Murai, K. Hara, R. Katoh, Effect of pH on absorption spectra of photogenerated holes in nanocrystalline $TiO_2$ films. Chem. Phys. Lett. **438**(4-6), 268-273 (2007). doi:10.1016/j.cplett.2007.03.017
